# Quantitative Visualization of the Nanomechanical Young’s Modulus of Soft Materials by Atomic Force Microscopy

**DOI:** 10.3390/nano11061593

**Published:** 2021-06-17

**Authors:** Seongoh Kim, Yunkyung Lee, Manhee Lee, Sangmin An, Sang-Joon Cho

**Affiliations:** 1 Park Systems Corporation, 109 Gwanggyo-ro, Yeongtong-gu, Suwon 16229, Gyeonggi, Korea; jake.kim@parksystems.com (S.K.); cathy.lee@parksystems.com (Y.L.); 2 Department of Physics, Chungbuk National University, Cheongju 28644, Chungbuk, Korea; mlee@cbnu.ac.kr; 3 Department of Physics, Institute of Photonics and Information Technology, Jeonbuk National University, Jeonju 54896, Jeollabuk, Korea

**Keywords:** atomic force microscopy, Young’s modulus, cantilever, stiffness, tip radius

## Abstract

The accurate measurement of nanoscale mechanical characteristics is crucial in the emerging field of soft condensed matter for industrial applications. An atomic force microscope (AFM) can be used to conduct nanoscale evaluation of the Young’s modulus on the target surface based on site-specific force spectroscopy. However, there is still a lack of well-organized study about the nanomechanical interpretation model dependence along with cantilever stiffness and radius of the tip apex for the Young’s modulus measurement on the soft materials. Here, we present the fast and accurate measurement of the Young’s modulus of a sample’s entire scan surface using the AFM in a newly developed PinPoint^TM^ nanomechanical mode. This approach enables simultaneous measurements of topographical data and force–distance data at each pixel within the scan area, from which quantitative visualization of the pixel-by-pixel topographical height and Young’s modulus of the entire scan surface was realized. We examined several models of contact mechanics and showed that cantilevers with proper mechanical characteristics such as stiffness and tip radius can be used with the PinPoint^TM^ mode to accurately evaluate the Young’s modulus depending on the sample type.

## 1. Introduction

Since its discovery, atomic force microscopy (AFM) [1] has become a widely used technique for the structural and mechanical characterization of materials at the nano-scale [2]. The AFM technique has several advantages, including its ease of use, high-resolution three-dimensional (3D) imaging, compatibility with a wide range of accessible samples (conductor, semiconductor, and insulator), and suitability with various measurement conditions (air, liquid, and vacuum). While AFM is well-known for imaging surfaces, it is also a powerful tool to investigate nanomechanical properties such as the modulus, adhesion, deformation, stiffness, and energy dissipation of materials [3,4,5,6,7,8].

About 20 years after the pioneering experiment, simultaneous measurement of elastic, electrostatic, and adhesive properties, called pulsed-force mode operation [9], there have been various types of nanoscale AFM imaging techniques for revealing the nanomechanical properties of soft materials based on the site-specific force spectroscopy. The PeakForce tapping mode of AFM (Bruker Co., Billerica, MA, USA) provides quantitatively characterized nanoscale materials which keep the maximum contact force with force–distance curve at every pixel position on the sample surface and thus allows it to image the surface topography and the interaction map simultaneously, such as nanomechanical and biological imaging of living cell [10,11]. Similarly, for the bimodal tapping mode of AFM (Oxford Instruments NanoAnalysis & Asylum Research Co., High Wycombe, United Kingdom), the cantilever is simultaneously driven at two eigenmode frequencies, which enables imaging of both topography and nanomechanical properties with a capability of high resolution and a wide range of Young’s modulus [12,13]. In addition, the Fast Force Mapping mode of AFM (Asylum Co., Santa Barbara, CA, USA) was introduced for overcoming time consumption, in which the tip–sample distance is modulated in a sinusoidal motion at up to 300 Hz. The Multifrequency AFM utilizes multiple eigenmodes of the cantilever’s resonance frequency to detect surface morphology as well as nanomechanical properties of the samples [14,15,16].

Although those techniques enable the measurement of novel nanomechanical information of the samples such as Young’s modulus, the measured results vary with the mechanical characteristics of the cantilever used and the contact mechanics model [17] employed. Accurate quantification of the Young’s modulus of sample surfaces is crucial for industrial applications involving soft matter such as polymers [18]. To accurately evaluate the Young’s modulus using AFM, it is important to use cantilevers with proper mechanical characteristics such as stiffness and tip radius. Several groups have demonstrated that even with the same indentation depth, cantilever tips with different radii yield inconsistent results for a sample’s nanomechanical properties, showing a dependence of the measured Young’s modulus on tip radius [19,20]. Moreover, the use of a sharp tip for measuring soft films can lead to inaccurately high values of the Young’s modulus due to tip penetration into the surface [21], including evaluating nanoindentation on stiffer substrates [22]. However, there is still a lack of well-organized study about the nanomechanical interpretation model dependence along with cantilever stiffness and radius of the tip apex for the Young’s modulus measurement on the soft materials.

Here, we comprehensively evaluated the Young’s modulus of soft matter samples using the PinPoint^TM^ nanomechanical mode of the AFM. The PinPoint^TM^ nanomechanical mode allows the measurement of topographical information and nanomechanical properties, including the Young’s modulus at each pixel within the entire scan area, thus simultaneously and quantitatively visualizing the topographical image and mapping the Young’s modulus of a sample’s surface. Using this AFM mode, we first examined three contact mechanics models, i.e., the Hertzian model [23], Derjaguin–Muller–Toporov (DMT) model [24], and Johnson–Kendall–Roberts (JKR) model [25,26], for analyzing the Young’s modulus of three types of polymers. Secondly, we showed the effect of cantilever stiffness on the measurement of the Young’s modulus. Finally, we checked how the cantilever’s radius affected the determination of the Young’s modulus. The study results demonstrate that it is essential to use cantilevers with proper stiffness and radius and to invoke an adequate contact mechanics model for accurate evaluation of the Young’s modulus depending on the sample type.

## 2. Materials and Methods

### 2.1. Instrument

We used the PinPoint^TM^ nanomechanical mode of an AFM system (Park Systems Co., NX10, Suwon, Korea) to gather high-resolution topographical images while simultaneously obtaining force–distance curves at each pixel of the entire scan area. The PinPoint^TM^ nanomechanical mode allows simultaneous visualization of surface morphology and quantitative nanomechanical properties such as modulus, adhesion, deformation, stiffness, and energy dissipation. All measurements were conducted in ambient air with the same experimental parameters: image size, 7 µm × 7 µm and 10 µm × 10 µm; pixels, 256 × 256; force–distance (FD) curves, 65,536; sample deformation, 4 nm~5 nm; and z-scanner speed, 25 μm/s.

### 2.2. Cantilever

To study the effect of cantilever stiffness on the Young’s modulus calculation, we used three cantilevers with different spring constants (or stiffness): PPP-CONTSCR (Nanosensors, Neuchatel, Switzerland) as the softest cantilever with a spring constant of 0.2 ± 0.03 N/m, PPP-FMR (Nanosensors, Switzerland) with a spring constant of 2.8 ± 0.2 N/m as a moderately hard cantilever, and PPP-NCHR (Nanosensors, Switzerland) with a spring constant of 42 ± 3 N/m as the hardest cantilever, as summarized in Table 1, which are calibrated by using the thermal noise method [27].

To study the dependence of tip radius on the determination of Young’s modulus values, we used two cantilevers, PPP-FMR (spring constant of 2.8 ± 0.2 N/m, tip radius of 10 nm) and SD-R30-FM (spring constant of 2.8 ± 0.3 N/m, tip radius of 30 nm), with the same resonance frequency, length, width, and thickness.

### 2.3. Samples

We investigated four polymer-based samples as follows: polydimethylsiloxane (PDMS) as an example of a soft polymer (Young’s modulus of 1 MPa~2 MPa), low-density polyethylene (LDPE, Young’s modulus of 100 MPa~200 MPa) as a moderately hard polymer, and polystyrene (PS, Young’s modulus of 1 GPa~3 GPa) as hard polymers. Each sample was mounted on a 12 mm steel sample disc with a flat and uniform surface to stabilize sample loading. Note that for the optical detection part for the cantilever displacement, we checked the sensitivity of the cantilever by using a force–distance curve. However, in the case of a hard sample such as PS, we obtained the slope value with a small pushing distance because the tip radius might be changed by pushing force to check the sensitivity of the slope of force value with respect to pushing depth.

## 3. Results and Discussion

### 3.1. Evaluation of Young’s Modulus Based on Contact Mechanics Models

**Figure 1:**
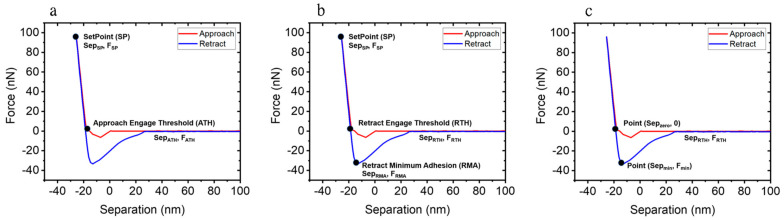
Experimental force–separation curves based on (**a**) the Hertzian model, (**b**) the DMT model, and (**c**) the JKR model.

Three representative contact mechanics models were considered for calculating the Young’s modulus values in our AFM-based measurements. These models play a key role in understanding contact mechanics between an AFM tip and a sample surface, with a specific emphasis on the deformation process of the sample surface in contact with the tip. Figure 1 shows the three contact mechanics models that were used.

The Hertzian model (Figure 1a) only considers elastic deformation such as contact between two elastic materials [23]. It disregards any adhesion phenomenon between the AFM tip and the sample surface. The Young’s modulus with the Hertzian model can be expressed as follows:

(1) $$E=\frac{3}{4}\frac{{\left(1-v\right)}^{2}}{r^{1/2}}{\left\{\frac{{\left(F_{SP}\right)}^{2/3}-{\left(F_{ATH}\right)}^{2/3}}{Sep_{SP}-Sep_{ATH}}\right\}}^{3/2},$$

where *E* is Young’s modulus, *ν* is Poisson’s ratio, *r* is the AFM tip radius, $F_{SP}$ and $Sep_{SP}$ are the force and the separation distance at the setpoint (*SP*), respectively, and $F_{ATH}$ and $Sep_{ATH}$ are the force and distance at the ‘approach engage threshold (*ATH*)’, respectively, as indicated in Figure 1a. Here, we obtained forces and separation points from FD curves and calculated the Young’s modulus with measured force and distance values using Equation (1).

The Derjaguin–Muller–Toporov (DMT) model (Figure 1b) can be applied to harder Young’s modulus > 1 GPa, and adhesive samples [24]. Unlike the Hertzian model, the DMT model incorporates adhesion between the tip and the sample surface into the Young’s modulus calculations as follows:

(2) $$E=\frac{3}{4}\frac{{\left(1-v\right)}^{2}}{r^{1/2}}{\left\{\frac{{\left(F_{SP}-F_{RMA}\right)}^{2/3}-{\left(F_{RTH}-F_{RMA}\right)}^{2/3}}{Sep_{SP}-Sep_{RTH}}\right\}}^{3/2},$$

where $F_{SP}$ and $Sep_{SP}$ are the force and the separation distance at the setpoint (*SP*), respectively, $F_{RTH}$ and $Sep_{RTH}$ are the force and the distance at the position of ‘retract engage threshold (*RTH*)’, and $F_{RMA}$ is the force at the position of ‘retract minimum adhesion (*RMA*)’, as indicated in Figure 1b.

The JKR model (Figure 1c) is well suited for soft materials with Young’s modulus < 1 GPa and with strong adhesion [25,26]. The main difference between the DMT and JKR models is the applicable range of adhesion force. While the DMT model considers long range adhesion forces (including before contact), the JKR model only takes adhesion forces from the point of contact into account. The Young’s modulus is expressed as follows:

(3) $$E=\frac{3}{4}{\left(1-v\right)}^{2}{\left\{\frac{1+16^{1/3}}{3}\right\}}^{3/2}\frac{F_{min}}{{\left(r{\left(Sep_{zero}-Sep_{min}\right)}^{3}\right)}^{1/2}},$$

where $F_{min}$ is the minimum force at *z* = $Sep_{min}$ and $Sep_{zero}$ is the distance where the force is zero during the retraction, as indicated in Figure 1c.

**Figure 2:**
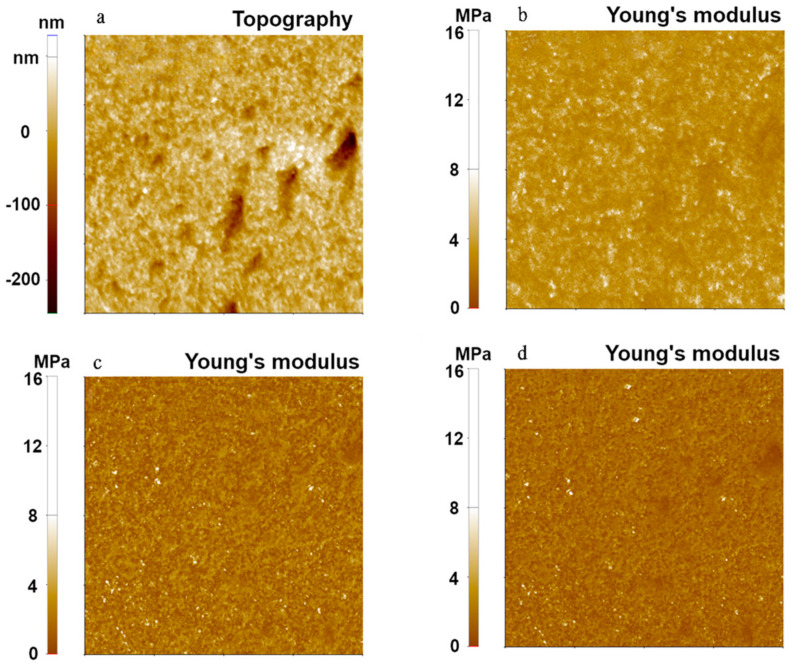
Surface topography (**a**) and Young’s modulus images (**b**–**d**) of PDMS using the PinPoint^TM^ nanomechanical mode. The Young’s modulus was calculated using the Hertzian model (**b**), the DMT model (**c**), and the JKR model (**d**). The scan size of all images was 10 μm × 10 μm.

To compare each contact model for different polymers, three polymer-based samples with different Young’s modulus (from 1 MPa to 2 GPa) were used to measure their mechanical properties using the FMR cantilever and the PinPoint^TM^ nanomechanical mode. Figure 2 shows topographical images and simultaneously measured Young’s modulus images of PDMS, the softest polymer sample, with a setpoint of 10 nN and *Z*-scanner speed of 25 μm/s, where Young’s modulus values were evaluated using the three models.

While Young’s modulus values obtained from the DMT and JKR models were similar (1.91 MPa from the DMT model, 1.20 MPa from the JKR model), the Hertzian model resulted in a relatively high value of Young’s modulus (6.47 MPa). In addition, the Hertzian model displayed a higher standard deviation (1.43 MPa from Hertzian) than the DMT model and the JKR model (0.87 MPa for DMT and 0.52 MPa for JKR). We found that both DMT and JKR models consistently led to the expected value of Young’s modulus, which was about 1~2 MPa for PDMS. The slightly elevated modulus from the DMT model was likely due to the softness of PDMS, which was in a suitable range so that JKR delivered more reliable values. On the other hand, the Hertzian model was shown to be unsuitable for the PDMS sample since it neglected the adhesion force that occurred on the sample, leading to erroneous calculations of the Young’s modulus.

**Figure 3:**
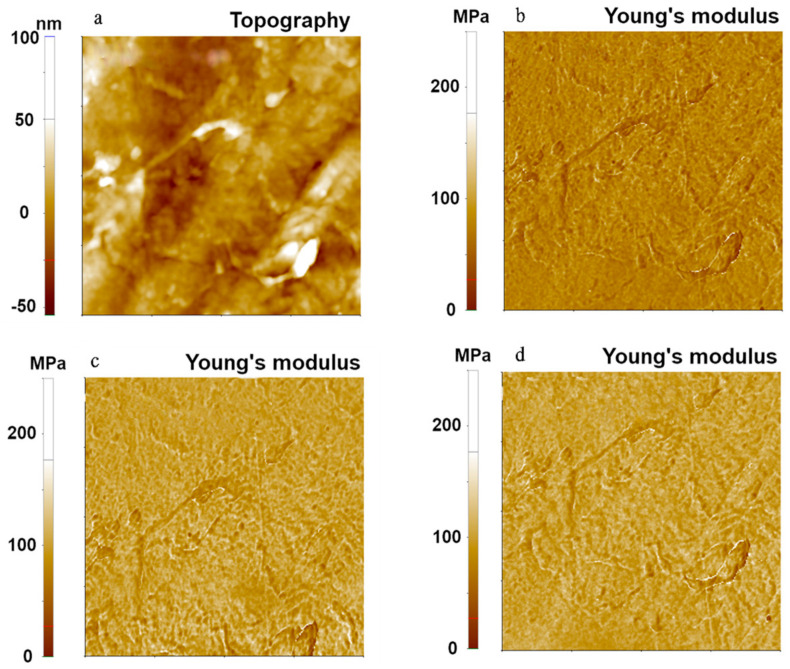
Topography (**a**) and Young’s modulus (**b**–**d**) images of LDPE using the PinPoint^TM^ nanomechanical mode. Young’s modulus images were obtained using Hertzian (**b**), DMT (**c**), and JKR (**d**) models. The scan size of all images was 10 μm × 10 μm.

We then examined the three contact mechanics models for a moderately hard polymer, LDPE, using the FMR cantilever. Results are shown in Figure 3. The topography and Young’s modulus images of LDPE were obtained with the same measurement parameters as in the experiment for the PDMS sample. Young’s modulus values of 74.94 MPa (Hertzian), 101.04 MPa (DMT), and 99.92 MPa (JKR) were obtained with a standard deviation of 13.66 MPa for the Hertzian model, 14.72 MPa for the DMT model, and 14.38 MPa for the JKR model. As with the PDMS sample, the Hertzian model gave a different value of the Young’s modulus from the values obtained with the other two models. This was due to the non-negligible adhesion force between the AFM tip and the LDPE sample surface. The Young’s modulus obtained using the DMT model consistently matched with that obtained with the JKR model. Note that the standard deviation differences of each model come from the spot variation of scanned position by lateral thermal drifting as a measurement limitation.

**Figure 4:**
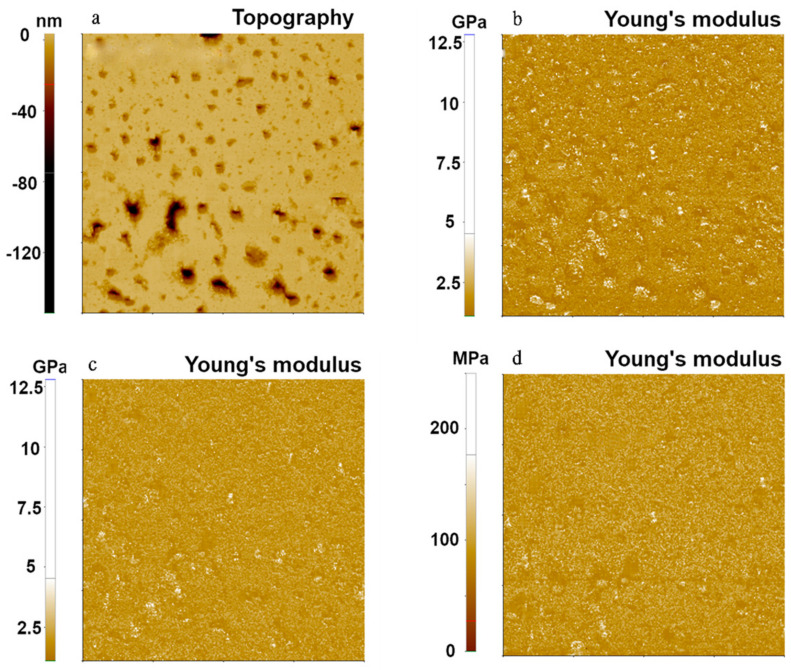
Topography (**a**) and Young’s modulus (**b**–**d**) images of PS using the PinPoint^TM^ nanomechanical mode. These Young’s modulus images were obtained using Hertzian (**b**), DMT (**c**), and JKR (**d**) models. The scan size of all images was 10 μm × 10 μm.

Figure 4 shows the topography and Young’s modulus images of PS, the hardest polymer sample using the FMR cantilever. They were taken using the same parameters as those used for the other two polymers. The calculated Young’s modulus values were 1.73 GPa, 1.41 GPa, and 1.40 GPa with standard deviations of 0.69 GPa, 0.28 GPa, and 0.33 GPa for Hertzian, DMT, and JKR models, respectively. The Hertzian model produced relatively higher values of Young’s modulus than the DMT and JKR models, and the DMT and JKR models showed similar values of Young’s modulus. This trend was also found in PDMS (Figure 2) due to adhesion forces occurring between the tip and the PS sample. All models delivered Young’s modulus values within the range of expected values (1–2 GPa).

**Figure 5:**
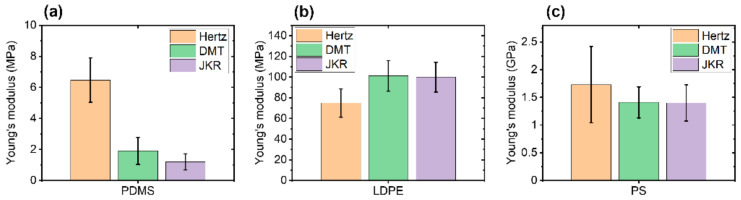
Young’s modulus for each polymer sample using different contact mechanics models in the case of PDMS (**a**), LDPE (**b**) and PS (**c**).

The Young’s modulus obtained using the Hertzian model showed consistent deviations from those obtained using the other two models for all samples as summarized in Figure 5. This was due to the adhesion force occurring between the tip and the polymer-based sample, which was neglected in the Hertzian model. Young’s modulus values with DMT and JKR models coincided well for LDPE and PS but showed a slight difference for the PDMS polymer. The difference found for the PDMS sample might have originated from the softness of the PDMS sample. Generally, the JKR model delivered more reliable results for Young’s modulus values below 1 GPa. Therefore, to accurately evaluate Young’s modulus values, one should use a mechanical contact model that is applicable to the sample being measured.

### 3.2. Effects of Cantilever Stiffness on Young’s Modulus Evaluation

**Figure 6:**
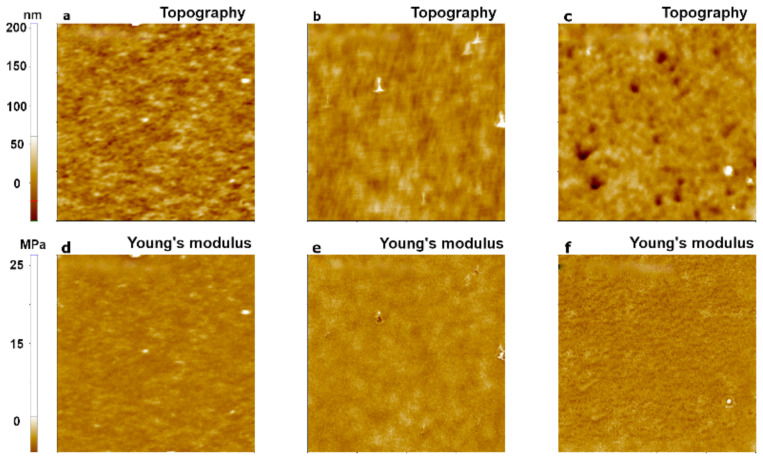
PinPoint^TM^ nanomechanical images of a PDMS sample using three AFM cantilevers with different spring constants: 0.2 N/m for CONTSCR (**a**,**d**), 2.8 N/m for FMR (**b**,**e**), and 42 N/m for NCHR (**c**,**f**). The scan size of all images was 10 μm × 10 μm.

To investigate the dependence of Young’s modulus measurements on cantilever stiffness, we performed an experiment with three cantilevers that had different stiffness values: CONTSCR, FMR, and NCHR (see Materials and Methods). We used the PDMS sample and analyzed data obtained with the JKR model. The JKR model is suitable for soft materials with Young’s modulus <1 GPa and strong adhesion [11]. Figure 6 shows the topography and Young’s modulus images of PDMS evaluated using the JKR model. CONTSCR and FMR cantilevers showed similar Young’s modulus values (1.32 MPa with CONTSCR and 1.53 MPa with FMR), while the NCHR cantilever showed a much higher value (28.12 MPa). CONTSCR and FMR cantilevers delivered good agreement with the expected modulus values (1–2 MPa), while the NCHR cantilever was not suitable for soft materials such as PDMS.

**Figure 7:**
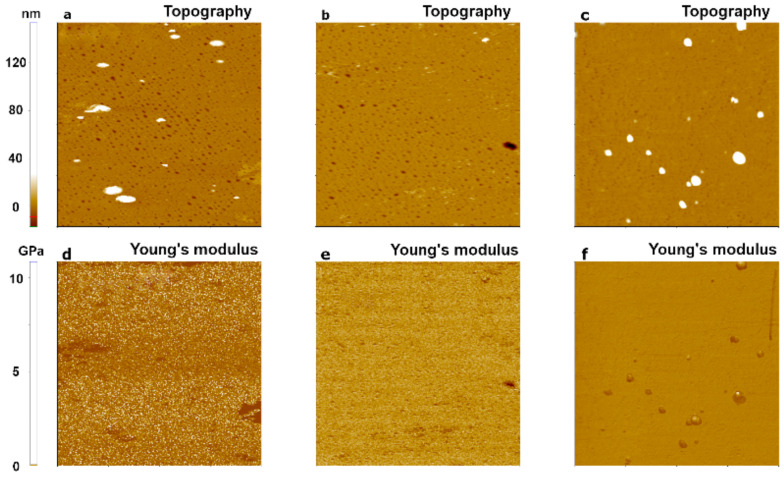
PinPoint^TM^ nanomechanical images of a PS sample using three cantilevers with different spring constants: 0.2 N/m for CONTSCR (**a**,**d**), 2.8 N/m for FMR (**b**,**e**), and 42 N/m for NCHR (**c**,**f**). The size of all images was 10 μm × 10 μm.

Figure 7 shows the topography and Young’s modulus images for a hard polymer sample, PS. FMR and NCHR cantilevers resulted in similar Young’s modulus values (FMR: 1.19 GPa, NCHR: 1.36 GPa). However, the CONTSCR cantilever resulted in a slightly elevated Young’s modulus value (2.90 GPa) along with a large standard deviation. Such a large standard deviation meant that measurements for this cantilever had a wider distribution range with reduced accuracy compared with the other two cantilevers. Note that previous measurement claimed nominal Young’s modulus value of block copolymer along with PS portion with PMMA as the range of 2~2.7 GPa recommended with usage of a stiff cantilever as a force constant of ~5 N/m or ~40 N/m [28,29,30].

**Figure 8:**
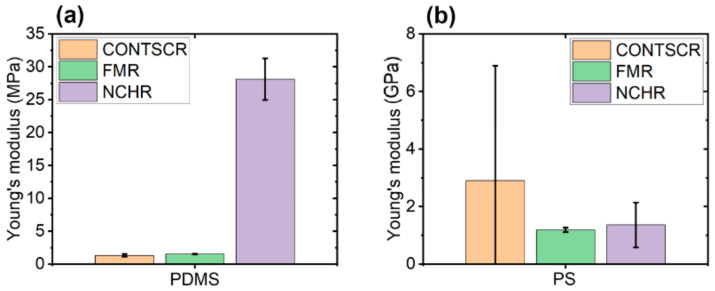
Young’s modulus for two polymer samples with respect to three different AFM cantilevers in the case of PDMS (**a**) and PS (**b**).

Figure 8 shows the comparison of two types of polymer samples (PDMS, PS) with respect to three different AFM cantilevers. As a result, one should consider choosing the appropriate cantilever stiffness for the measurement of Young’s modulus with respect to sample hardness character, e.g., using hard cantilevers with hard samples and soft cantilevers with soft samples.

**Figure 9:**
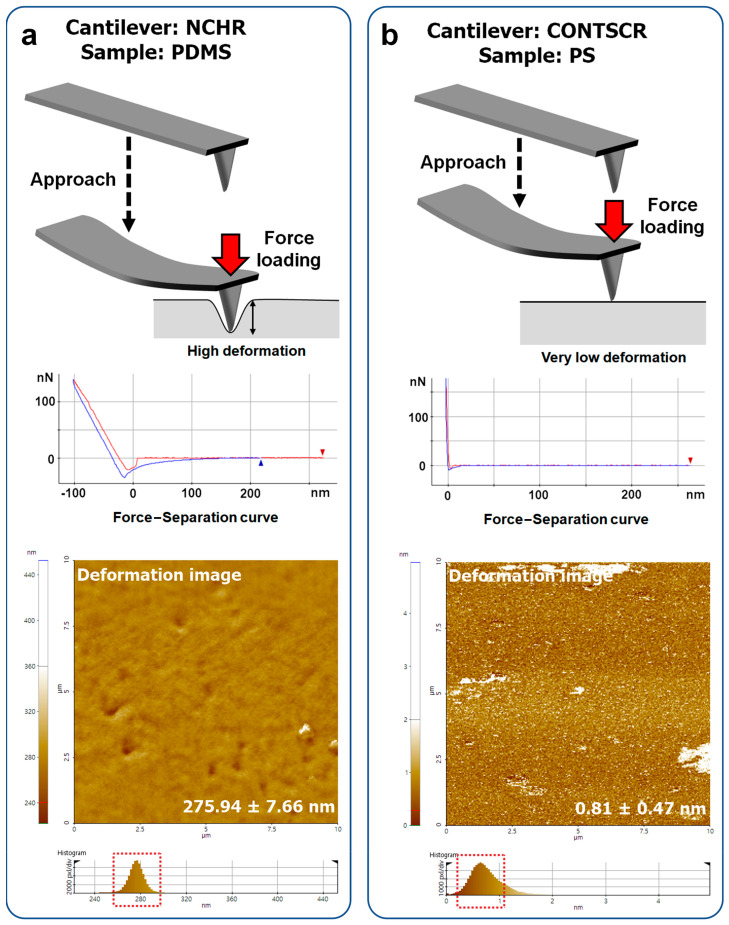
Sample deformation occurring during FD curve measurements. (**a**) High deformation when using a hard cantilever–soft material (NCHR–PDMS) and (**b**) very small deformations when using a soft cantilever–hard material (right, CONTSCR–PS).

For example, a cantilever with a high spring constant yielded inaccurate results for soft samples (~276 nm of deformation, Figure 9a) due to a strong loading force and an undefined contact area. On the other hand, on hard materials (>1–2 GPa), a soft cantilever did not induce sufficient deformation (~0.8 nm of deformation, Figure 9b). Thus, the measured Young’s modulus values showed a large standard deviation and inaccuracy. The very small deformation resulted in unintended signals such as noisy spots in Figure 7d, as evidenced in the Young’s modulus image using the CONTSCR cantilever (white spots, sometimes higher than 1 TPa).

For a soft material (PDMS), cantilevers with small or moderate spring constants (CONTSCR, FMR) yielded consistently accurate results with acceptable standard deviations. However, a cantilever with a high spring constant gave an inaccurate result with an elevated standard deviation due to a large loading force and subsequently increased deformation. For a hard material (PS), accurate results were obtained with hard and moderate cantilevers (NCHR, FMR). By contrast, the CONTSCR tip yielded poor results with unexpected error signals due to insufficient loading force and deformation. Therefore, appropriate cantilever selection is essential for measuring Young’s modulus values, depending on the sample type.

### 3.3. Effects of Tip Radius on Young’s Modulus Evaluation

**Figure 10:**
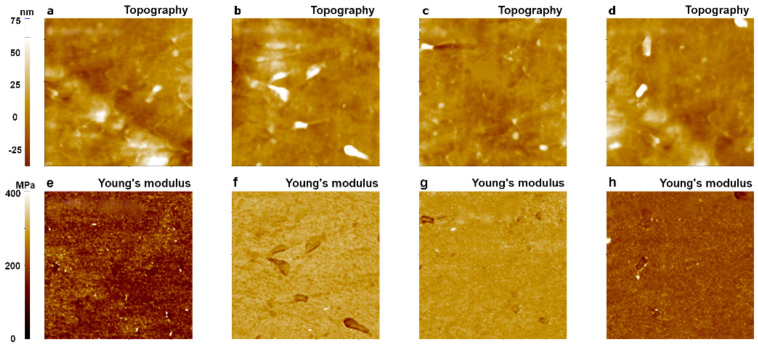
Topography and Young’s modulus images of LDPE obtained with a PPP-FMR cantilever (tip radius: 10 nm) using the PinPoint^TM^ nanomechanical mode of AFM. The 1st image (**a**,**e**); 10th image (**b**,**f**); 100th image (**c**,**g**); and 200th image (**d**,**h**). The size of all images was 10 μm × 10 μm.

The topography and Young’s modulus values of an LDPE sample were imaged 200 times using a single PPP-FMR cantilever with a tip radius of about 10 nm over different sample surfaces. From 200 images that showed similar results, we randomly picked four images for statistical analysis of the Young’s modulus values. As shown in Figure 10, Young’s modulus images shown in Figure 10a,d revealed proper Young’s modulus values of LDPE in a range of 100 MPa to 200 MPa, while images shown in Figure 10b,c displayed higher values than expected (368 MPa–387 MPa).

**Figure 11:**
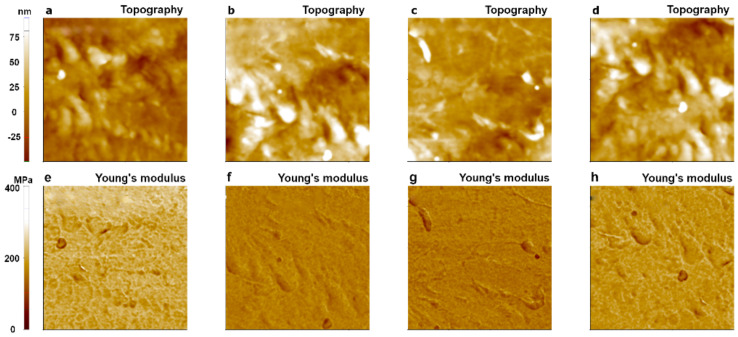
Topography and Young’s modulus images of LDPE obtained with an SD-R30-FM cantilever (tip radius: 30 nm) using the PinPoint^TM^ nanomechanical mode of AFM. The 1st image (**a**,**e**); 10th image (**b**,**f**); 100th image (**c**,**g**); and 200th image (**d**,**h**). The size of all images was 10 μm × 10 μm.

The same measurements were performed using a different tip of SD-R30-FM (tip radius of ~30 nm) with the same conditions used for the PPP-FMR case. All measured Young’s modulus values fell into the expected range for LDPE (100~200 MPa) even after 200 imaging cycles, indicating superior wear resistance of the sharp tip in the PinPoint^TM^ nanomechanical mode as shown in Figure 11. This implies that the use of a tip with a large radius is suitable for measuring the Young’s modulus value of LDPE, a soft matter, resulting in minor fluctuations of the measured values compared to the modulus values obtained with a small radius tip (Figure 10).

**Figure 12:**
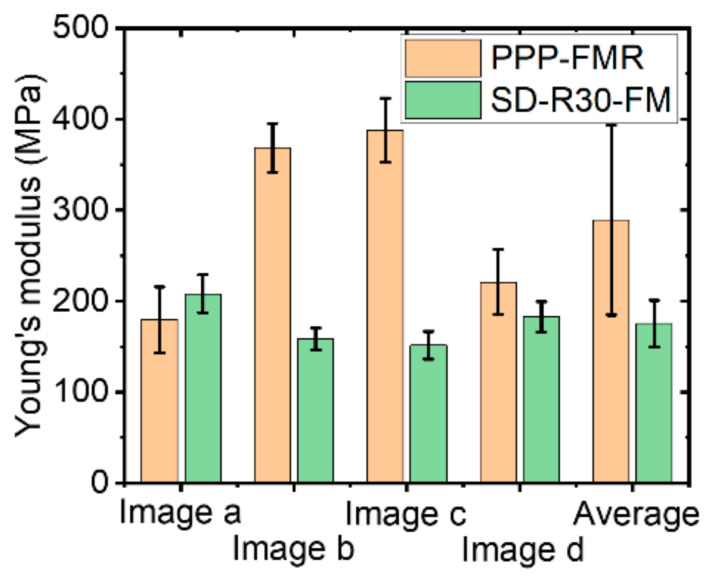
Young’s modulus of an LDPE sample with different AFM tip radii.

To calculate the exact modulus value, it is important to clearly define the AFM tip end geometry and the contact area of the sample surface. However, if the tip end becomes dull during the measurement, or if the contact area between the tip and the sample with a rough surface has a different value than expected, these factors related to undefined indentation might lead to inaccurate results. Results of 200 measurements with one single sharp tip indicated highly fluctuating values of the tip-sample contact area during imaging, leading to large fluctuations of the Young’s modulus values with large standard deviations (Figure 12). The trend of calculated Young’s modulus values for SD-R30-FM cantilevers appeared to be reliable, and the total average was well fitted in the proper range (100 MPa~200 MPa) with a small standard deviation. The use of a cantilever with a large radius tip showed the expected value of the Young’s modulus for the bulky glassy polymer sample LDPE, whereas the use of a sharp tip gave large fluctuations for Young’s modulus values. Note that we expect soft sample (PDMS) shows more higher value differences of Young’s modulus with respect to the cantilever tip sharpness because the sharp cantilever can penetrate more in the soft sample, while the hard PS sample shows less value differences of Young’s modulus.

We evaluated the Young’s modulus values with cantilevers having different tip radii using the PinPoint^TM^ nanomechanical mode of AFM. To examine the effect of tip radius, multiple tests with the same sample were performed over different positions on the sample surface. Using a sharp tip with a small radius of 10 nm, we measured largely fluctuating values of the Young’s modulus with large standard deviations. On the other hand, using a large radius tip of about 30 nm, we obtained consistent values of the Young’s modulus with negligible fluctuations. This indicates that the relatively wide contact area between the tip and the surface allows appropriate evaluation of Young’s modulus via the collective effect of molecular level elastic response on the surface; on the contrary, a sharp tip causes a problem in elastic force measurement due to penetration on the surface. Although one can perform stable measurements with a large tip rather than with a small sharp tip, the use of a sharp tip generally offers high-resolution imaging. Therefore, it is important to use a cantilever with a proper radius to accurately determine nanomechanical properties including Young’s modulus depending on the sample type.

## 4. Conclusions

In this study, we measured the Young’s modulus values of various soft and hard polymeric materials at each pixel within the entire scan area, from which the quantitative visualization of the mechanical property was successfully implemented. The present method with the PinPoint^TM^ nanomechanical mode not only enables direct visualization of the Young’s modulus in addition to surface topography, but also allows statistical analysis of measured values at each pixel. Through statistical analysis, we could unambiguously determine the appropriate measurement conditions and relevant mechanics model such as tip radius and stiffness for each sample, thus allowing an accurate evaluation of Young’s modulus. Following previous works to take open mark out for appropriate usage of cantilever for measurement of Young’s modulus, we certainly showed a well-organized definition of the suitability of the cantilever choice with accurate quantitative evaluation of the target samples’ Young’s modulus. Those techniques enable the measurement of novel nanomechanical information of the samples such as Young’s modulus. The measured results vary with the mechanical characteristics of the cantilever used and contact mechanics model. We found that the JKR model and a soft cantilever were suitable for measuring the Young’s modulus of soft/medium materials with a modulus <1 GPa with strong adhesion, while a moderately hard cantilever was more suitable for relatively hard materials with a modulus of roughly >1 GPa. In addition, the use of a large tip with a radius of about 30 nm provided more reliable and consistent results than the use of a relatively sharp tip. This indicates that the relatively wide contact area between the tip and the surface allows appropriate evaluation of Young’s modulus via the collective effect of molecular level elastic response on the surface, while on the contrary, a sharp tip causes a problem in elastic force measurement due to penetration on the surface. Therefore, it is important to use a proper cantilever and an adequate analysis model to accurately determine nanomechanical properties such as the Young’s modulus of soft materials.

## Figures and Tables

**Table 1:** Specifications of three cantilevers used for evaluating the effect of cantilever stiffness on Young’s modulus calculations.

	Spring Constant (N/m)	Resonance Frequency (kHz)	Length (μm)	Width (μm)	Thickness (μm)
PPP-CONTSCR	0.2 ± 0.03	25	225	48	1
PPP-FMR	2.8 ± 0.2	75	225	28	3
PPP-NCHR	42 ± 3	330	125	30	4

## Data Availability

Data sharing not applicable.
